# Population well-being and electoral shifts

**DOI:** 10.1371/journal.pone.0193401

**Published:** 2018-03-12

**Authors:** Jeph Herrin, Dan Witters, Brita Roy, Carley Riley, Diana Liu, Harlan M. Krumholz

**Affiliations:** 1 Section of Cardiovascular Medicine, Yale School of Medicine, New Haven, Connecticut, United States of America; 2 The Gallup Organization, Washington, DC, United States of America; 3 Department of Pediatrics, University of Cincinnati College of Medicine, Cincinnati, Ohio, United States of America; 4 Division of Critical Care Medicine, Department of Pediatrics, Cincinnati Children’s Hospital Medical Center, Cincinnati, Ohio, United States of America; Iowa State University, UNITED STATES

## Abstract

Population wellbeing, an aggregate measure of positive mental, physical, and emotional health, has previously been used as a marker of community thriving. We examined whether several community measures of wellbeing, and their change since 2012, could be used to understand electoral changes that led to the outcome of the 2016 United States presidential election. We found that areas of the US which had the largest shifts away from the incumbent party had both lower wellbeing and greater drops in wellbeing when compared with areas that did not shift. In comparison, changes in income were not related to voting shifts. Well-being may be more useful in predicting and understanding electoral outcomes than some more conventional voting determinants.

## Introduction

The 2016 United States Presidential election was determined, in part, by areas of the country that shifted support to the Republican candidate. Efforts have been made to characterize the areas with the most shift, with prior reports identifying the areas as having higher levels of alcohol and opioid use [1], being less healthy [2,3], having rising levels of unemployment [4], and lower rates of college education [5]. However, to date, studies have not assessed the association of well-being, a metric of subjective experience of life that includes elements of physical, social, mental and emotional health, nor its recent change over time, with voting patterns. Prior research in other contexts has found a positive relationship between well-being and a voting preference for incumbents, independent of economic measures [6,7], and it is plausible that poor and worsening well-being was associated with a vote to change the party of the President. Accordingly, we tested the hypotheses that population well-being and four year change in population well-being were associated with shifts in voting preference regarding the incumbent party.

## Methods and materials

### Voting shifts

Our primary unit of analysis was the county (or county equivalent), which was the smallest geographic unit for which we could obtain both voting results and well-being data. We used voting results which are available from U.S. Voting Atlas for a small fee [8]; our analysis included voting results from every county (or county equivalent) in the U.S. except for those in the state of Alaska, which were not available for the 2016 election at the county level as of time of analysis [8]. For each county we used the percentage of votes earned by the Republican nominee in each year, using all votes (i.e., including third party candidates) as the denominator. The voting shift was calculated as the change in percentage vote for the Republican nominee from 2012 to 2016.

The well-being survey data were collected using a complex stratified survey design. This design precludes direct aggregation of survey responses to create area measures; such summary measures would not account for the different patterns of response across different counties. To compensate for disproportionalities in selection probabilities and nonresponse, we post-stratified the well-being data for each zip code grouping using an iterative proportional fitting (i.e., raking) algorithm to account for nonrandom nonresponse by phone status (land line or mobile), age, sex, region, education, population density, ethnicity, and race. Targets used for the weighting leveraged the most current data available from the Current Population Survey administered by the U.S. Census Bureau. This ‘reweighting’ using iterative proportional fitting is labor intensive and inappropriate for small sample sizes, including most individual counties in the U.S. Therefore, in order to link the voting shift to the well-being survey data, we categorized counties into a small number of groups according to their voting shift rate; this allowed us to construct accurately weighted estimates of well-being metrics for groups of counties with adequate numbers of survey respondents. Specifically, the voting shift was used to categorize U.S. counties into 6 groups, according to the percentage point shift: less than -10 (that is, greater than 10 percentage point shift toward the Democratic nominee), -10 to -5, -5 to 0 (inclusive), 0 to 5, 5 to 10, and more than 10 percentage point shift toward the Republican nominee.

### Well-being

To measure well-being, we used items from the Gallup-Healthways Well-Being Index, a nationally representative, geo-coded random digit dial outbound telephone survey for which 353,561 respondents were interviewed throughout the year in 2012 and 177,192 throughout the year in 2016. Though the full survey instrument includes items assessing many aspects of well-being, the Well-Being Index itself was significantly modified beginning in 2014; thus, for this analysis we report only on a subset of items which were included in both the 2012 and 2016 versions of the survey.

There were 10 items collected in both 2012 and 2016 related to well-being that we report here. Two items are Cantril’s Ladder [9], which asks respondents to consider their life as placed on a ‘step’ between 0 and 10 of a ‘ladder’, with the bottom step signifying the worst possible life for them and the top step signifying the best possible life for them; the first of these items asks respondents which step they think they are currently on, while the second asks them which step they think they will be on in 5 years. In line with the empirically determined reporting guidelines for these items, the first was reported as the percentages of respondents placing themselves on steps 0–4 and 7–10, while the second was reported as the percentages of respondents placing themselves on steps 0–4 and 8–10 [10]. One item asks respondents if they are satisified with the city or area where they live; this we report as the percent answering yes (versus no, don’t know, or refused). The remaining 7 items ask respondents if, for “a lot” of the day prior, they felt or expressed: happiness, stress, enjoyment, worry, smiling, sadness, and anger. These were also reported as the percentage of respondents in each shift category who answered “yes” (versus no, don’t know, or refused).

### Demographics

Because income, race, and education have been suggested as factors in the voting shifts for the 2016 election, we obtained data on these variables from the 2011 and 2015 U.S. Census for each county. Measures obtained were median household income, percent white race, and percent college graduates [11,12].

### Analysis

We summarized the number of counties, number of voters, median household income in 2015, change in median income from 2011, percent white, change in percent white, percent college educated and change in percent college educated for each of the shift categories, using non-parametric tests of trend to assess whether the last six factors were associated with voting shift category. Next, we calculated well-being scores for shift categories: the Gallup-Healthways Well-Being Index survey is conducted using a stratified survey design, so that design weights must be used to construct estimates for each category. In addition, to account for the imperfect randomness of each sample, survey responses were reweighted by voting shift group using iterative proportional weighting to produce demographically appropriate estimates. Thus, we calculated uniquely weighted responses for each of the six voting shift category regions for both 2012 and 2016.

Using these scores, we looked at two different relationships between well being and voting shift. First, for each well-being metric, we used a non-parametric test of trend to assess whether there was a trend over categories. Finally, we calculated the change in well-being for the same regions from 2012 to 2016, and again assessed for trend over shift categories. We report the P-values for the tests of trend.

## Results

### Shift categories

The regions comprising each shift category are reported in Table 1. Though lower household income in 2015 was associated with greater shift to the Republican nominee, neither the absolute nor the relative change in household income since 2011 followed the same trend; notably, the highest relative increase in household income occurred in the group of counties which showed the strongest shift *to* the Republican nominee, followed by the group of counties which shifted most *away* from the Republican nominee. Percentage of population that was white and the change in this percentage over four years were also not associated with shift category, though the rate of college education as well as the change in the rate of college education over four years both decreased with increasing voting shift (P = 0.025 for both).

**Table 1:** Income, race, and education. [source: US Census American Community Survey].

Change in Republican vote (%)	Counties	Respondents		Median Household Income			Race	Change from 2011	Education	Change from 2011
		2012	2016	2015	Change from 2011	% Change from 2011	2015% White		2015% College	
(min,-10]	46	8328	3871	$ 79,840.86	$ 2,820.41	3.7	80.5	-1.3	45.4	1.6
(-10,-5]	140	83070	44232	$ 66,222.64	$ 1,855.36	2.9	69.6	-0.2	38.9	1.6
(-5,0]	490	117235	58246	$ 57,470.10	$ 966.17	1.7	71.6	-0.7	32.4	1.5
(0,5]	1257	95731	47327	$ 50,583.18	$ 948.31	1.9	75.5	-0.5	24.3	1.4
(5,10]	851	37509	17970	$ 46,688.42	$ 1,281.87	2.8	89.4	-0.5	18.9	1.2
(10,max]	328	10780	4976	$ 43,660.68	$ 1,688.76	4.0	93.8	-0.5	15.6	1.0
P-value*				0.025	0.277	0.848	0.142	0.565	0.025	0.025

* P-value based on non-parametric test for trend.

### Well-being

The 2016 well-being responses are summarized in Table 2 according to voting shift category. The pattern of responses was consistent for each item with areas of increasing shift towards the Republican nominee reporting: lower percentage of respondents placing themselves on the top of the Cantril ladder, both currently and in 5 years; higher percentage placing themselves on the bottom, both currently and in 5 years; and less satisfaction with the city or area where they live. These trends were significant, as were negative trends in reported happiness, enjoyment, and smiling/laughter on any given day, and increased reported sadness. There was no significant trend in stress, worry, or anger.

**Table 2:** Well-being in 2016. Based on surveys of 177,192 respondents in 2016. [source Gallup-Healthways Well-being survey].

Change in Republican vote (%)	Current Life Satisfaction		Anticipated Life Satisfaction in 5 Years		Satisfied with where you live	Experienced a lot yesterday						
	% (0–4)	% (7–10)	% (0–4)	% (8–10)		Happy	Stress	Enjoyment	Worry	Smile	Sadness	Anger
**(min,-10]**	3.4	72.5	4.5	71.7	91.3	90.8	43.0	86.7	31.6	82.0	16.1	13.9
**(-10,-5]**	4.4	69.2	4.3	69.1	87.3	88.9	39.6	85.0	29.7	82.3	17.5	14.5
**(-5,0]**	4.9	66.9	5.1	67.2	85.1	88.7	40.0	85.0	29.3	81.3	17.2	14.5
**(0,5]**	6.0	63.5	6.2	63.6	83.5	87.8	39.1	84.0	29.6	79.9	18.3	14.5
**(5,10]**	6.4	61.8	7.4	59.9	83.7	87.9	40.5	84.1	29.0	78.4	18.2	14.5
**(10,max]**	7.1	60.9	7.7	57.9	82.9	86.5	40.4	83.1	29.5	77.1	18.6	13.2
P-value*	<0.001	<0.001	<0.001	<0.001	<0.001	<0.001	0.410	<0.001	0.114	<0.001	<0.001	0.932

* P-value based on a non-parametric test for trend over voting shift categories.

The changes in well-being from the same areas of the U.S. from 2012 to 2016 are shown in Table 3. The two largest shift categories (areas of the country where at least 5% of the vote shifted to the Republican candidate) had an increase in those reporting being on the ‘bottom’ of the Cantril ladder (versus a decrease for all areas which shifted away from the Republican candidate), and reported the smallest anticipated changes in their position for 5 years hence. These changes in current and future life evaluation, at the top and the bottom of the ladder, were all significantly associated with voting shift; for satisfaction with one’s city and all 7 affect items, there were no significant trends.

**Table 3:** Change in well-being, 2012–2016. Based on surveys of 353,561 and 177,192 respondents in 2012 and 2016 respectively. [source Gallup-Healthways Well-being survey].

Change in Republican vote (%)	Current Life Satisfaction		Anticipated Life Satisfaction in 5 Years		Satisfied with where you live	Experienced a lot yesterday						
	% (0–4)	% (7–10)	% (0–4)	% (8–10)		Happy	Stress	Enjoyment	Worry	Smile	Sadness	Anger
**(min,-10]**	-0.4	2.7	0.5	3.0	-0.4	-0.4	-0.4	-1.0	-1.1	-1.7	0.7	1.2
**(-10,-5]**	-0.2	2.0	-0.2	3.0	-0.1	0.2	-0.6	-0.3	-2.4	-1.7	-0.2	0.4
**(-5,0]**	-0.4	2.1	-0.1	2.3	0.2	0.4	0.0	0.1	-2.3	-1.5	-0.6	0.6
**(0,5]**	0.1	1.2	-0.1	2.0	0.1	0.2	-0.2	-0.1	-1.8	-1.9	0.3	0.6
**(5,10]**	0.3	0.8	0.0	1.8	-0.8	-0.4	0.7	-0.2	-2.1	-2.6	0.3	1.2
**(10,max]**	0.7	1.7	-0.5	1.6	-0.5	-1.2	0.1	-0.8	-2.2	-2.6	-0.7	-0.8
P-value*	0.001	<0.001	0.022	0.040	0.523	0.209	0.050	0.562	0.284	0.368	0.110	0.295

* P-value based on a non-parametric test for trend over voting shift categories.

There are several limitations to this study. First, as an observational study it is not possible to draw causal inferencess; any observed associations between well-being and voting shifts may be due to some unobserved factor. And there is no evidence that the people surveyed voted, or, if they did, if they voted differently than they did four years earlier. However, most examinations of voting determinants have these same limitations, and we believe that these findings offer important insights into the causes and implications of the overall election outcome. More specific to this study, we were constrained by our data to looking at very large groups of counties in order to construct population estimates of well-being; however, this constraint also means that our observed associations are true across large, diverse, heterogeneous groupings of counties, and thus more robust than a study examining a smaller or more homogeneous population. Finally, given our design, we were unable to adjust for potential effect modifiers; however, our estimates of well-being were standardized for demographic, socio-economic and population factors, accounting for much of heterogeneity known to relate to voting. While this topic surely deserves additional study, the current analysis is the largest and most representative examination of the relationship between well-being and voting patterns to date.

## Summary

These findings build on earlier work which found subjective well-being positively associated with electoral support for incumbents by linking decline in subjective well-being to decline in electoral support for the incumbent party.

These findings are limited by the observational study design, and open to ecological fallacy because there is no evidence that the people surveyed voted, or, if they did, if they voted differently than they did four years earlier

In conclusion, we suggest that multidimensional measures of population well-being may be important factors in electoral shifts and outcomes in the U.S., and that changes in population well-being may be a particular indicator of shifts in voter support. Focusing on well-being might serve incumbents well.
